# Antitetanic Serum

**Published:** 1904-08

**Authors:** 


### Abstracts and Selections.

### Antitetanic Serum.

It has been shown that guinea pigs and other animals inoculated with the poison of tetanus survive when treated at once with antitetanic serum. In France Nocard observed 375 animals of various kinds, all of which had been wounded, accidentally or surgically, and subjected to tetanic infection. These animals were given antitetanic serum at once, before the disease had time to develop. As a result not a single case of tetanus occurred among them. On the other hand, he noted fifty-five traumatized animals that had been exposed to tetanic infection, every one of which developed the disease.

In the August, 1899, number of *Medicine,* Prof. Geo. F. Butler, M. D., says: "Dr. Joseph Hughes, one of the most eminent and conservative veterinary surgeons in Chicago, has used the serum as a prophylactic in over five hundred cases following wounds, both surgical and accidental." Not a single case of tetanus has developed, though Dr. Hughes has used the serum where by former experience he was justified in expecting the disease to manifest itself.

From this and similar reports it has been proposed to immediately inject antiseptic serum in every case of traumatism of a suspicious character, hoping in this manner to prevent the subsequent development of tetanus. The serum is harmless to man, and may be given hypodermatically as the other serums. Nocard recommends that a first injection of ten cubic centimeters should be made as soon as possible after traumatism. A second injection should follow in from twelve to fifteen days.

It has been suggested to inject prophylactically all new-born infants in certain sections of Europe in which trismus neonatorum prevails.

In the *Therapeutic Gazette* for February 15, 1903, the editor directs attention to the fact that "although tetanus is, comparatively speaking, a rare disease, it is sufficiently frequent and fatal to make an antitetanic serum a much sought for remedy." He also pointed out the fact that the failure of antitetanic serum depended "not upon the fact that it was possessed of no virtue, but rather because it was used too late to combat the disease." The same writer expresses the view that "one fact stands out above all others, and that is, that thoroughly good results can not be expected from antitetanic serum unless it be given in the very earliest stages of the infection. So true is this that experienced observers have insisted that its best results can be obtained only when it is administered immediately after exposure to infection, without waiting until the micro-organisms have had a chance to develop in the body and produce early symptoms of poisoning.

The editor of the *New York Medical Journal,* in the issue of March 12, 1904, remarks that "the present drift of opinion seems to be to the effect that tetanus antitoxin, while probably of considerable prophylactic efficacy is of little use as a curative agent." At a meeting of the Paris Society of Surgery, according to the same editorial, M. Labbe expressed the view, that since the injection of antitetanic serum has been employed as a routine prophylactic measure, the disappearance of tetanus after surgical operations in horses was a prime fact in support of its preventive efficiency.

Furthermore, recent experience in the immediate topical employment of antitoxin in cases of toy pistol injuries appears to support our trust in its prophylactic value.

Bazy (Bulletins et Mêmoires de la Société de Chirurgie de Paris, 1896, N. S., XXII, 186, 191) had four cases of tetanus develop in his wards. From that period he applied preventive treatment to all cases of wounds admitted to his service. He made twenty-one preventive inoculations of ten cubic centimeters each. None of these patients developed tetanus, although he says their wounds belonged to that catagory which includes most cases of the disease.

*Dr. Joseph McFarland, in the Journal of the American Medical Association* for July 4, 1903, reports the results of a series of observations upon eight hundred horses which illustrate the value of antitetanic serum as a prophylactic agent. During a period of four years there had been a death rate of 10 per cent from tetanus, in spite of all precautions. A systematic immunization with antitetanic serum was then begun. Injections of ten to twenty-five cubic centimeters of serum were given every three months. As a result the death rate from tetanus rapidly decreased, and in the second year has been reduced to less than 1 per cent. The author believes that the practical conclusions to be drawn from these observations may be applied to the human subject. He thinks that antitetanic serum should be given as a prophylactic measure in all cases of suspicious wounds that are likely to be followed by tetanus.

Experiments made on guinea pigs by the author demonstrated that the dried serum fully protects inoculated animals.

At the twenty-ninth annual meeting of the Mississippi Valley Medical Association, held at Memphis, Tenn., October 7, 8 and 9, 1903, Dr. S. C. Stanton, of Chicago, contributed a valuable statistical paper on "The Prophylaxis of Tetanus" (*The Medical News,* October 31, 1903, page 860). Among the various prophylactic measures recommended by the author were the open treatment of all wounds, however insignificant, in which from the nature or surroundings there was any risk of tetanus, the immediate use of antitetanic serum in all cases of Fourth of July wounds, wounds received in barnyards, gardens, or other places where the tetanus bacillus was likely to be present, or tetanus infection likely to occur.

